# A pilot study investigating the effectiveness, appreciation, and feasibility of a cognitive stimulation program in dementia patients: online versus face-to-face

**DOI:** 10.3389/fpsyg.2025.1561157

**Published:** 2025-04-07

**Authors:** Simona Cintoli, Giulia Spadoni, Valeria Giuliani, Valentina Nicoletti, Eleonora del Prete, Daniela Frosini, Roberto Ceravolo, Gloria Tognoni

**Affiliations:** ^1^Neurology Unit, Integrated Assistance Departments (DAI) Neuroscience, Azienda Ospedaliero Universitaria Pisana (AOUP), Pisa, Italy; ^2^Department of Clinical and Experimental Medicine, University of Pisa, Pisa, Italy

**Keywords:** cognitive stimulation, non-pharmacological therapies, tele-rehabilitation, dementia, cognitive decline, online intervention, internet-based

## Abstract

**Introduction:**

This study was conducted to assess the feasibility, effectiveness, and appreciation of a cognitive stimulation protocol for dementia patients, comparing in-person and remote interventions. Cognitive stimulation is a key non-pharmacological therapy that supports cognitive abilities and psychological wellbeing in dementia patients, also benefiting caregivers. The COVID-19 pandemic highlighted the need for remote therapeutic options, yet the effectiveness and applicability of these for frail dementia patients require validation. The study aimed to evaluate whether a cognitive stimulation protocol could be adapted for remote use, particularly for patients facing logistical challenges.

**Methods:**

The study involved 19 dementia patients (Clinical Dementia Rating = 1 or 2), with 12 undergoing in-person treatment and seven participating remotely. Over eight weekly 1 h sessions, patients engaged in various cognitive activities, including memory, attention, and problem-solving exercises, guided by a clinical psychologist. Remote participants received an introduction to basic computer literacy. Assessments were conducted using the Mini-Mental State Examination (MMSE), Activities of Daily Living (ADL), and Instrumental Activities of Daily Living (IADL) at the start (T0) and end (T1) of the intervention. Additionally, satisfaction was measured with visual and Likert scales for both patients and caregivers.

**Results:**

No significant differences were found between the in-person and remote groups in terms of age, education, or gender. The cognitive profiles and ability to perform daily activities remained stable throughout the intervention. Both patients and caregivers reported high levels of satisfaction, with positive feedback on the utility, enjoyment, and engagement in the sessions. The program was also found to be effective in offering support and engaging caregivers, demonstrating that the protocol was both feasible and well-received.

**Discussion:**

These findings suggest that remote cognitive stimulation interventions are a viable and beneficial alternative to in-person therapy. The high levels of satisfaction and stable cognitive outcomes are in line with previous studies. Future research with a larger sample size and long-term follow-up is needed to further assess the lasting impact on cognitive function, quality of life, and caregiver burden. The integration of remote protocols into healthcare systems could enhance access to therapy for a broader patient population.

## 1 Introduction

Dementia, a broad category of cognitive impairments affecting memory, thinking, and behavior, is a leading cause of disability among older adults worldwide (World Health Organization, 2021). According to the World Health Organization (WHO), in 2019, approximately 55 million people were affected by dementia worldwide, with a projected increase to 78 million by 2030 (Li et al., 2024; GBD, 2022).

In Italy, as worldwide, dementia is one of the leading causes of disability among older adults, significantly impacting the quality of life of both patients and their caregivers. There are an estimated 1,126,961 cases of dementia in individuals aged 65 and older, as well as 23,730 cases of early-onset dementia in individuals aged 35–64 (ISS, 2024).

Managing dementia requires a multidisciplinary approach that includes early diagnosis, pharmacological and non-pharmacological interventions, psychological support for the patient-caregiver dyad, and social care. In recent years, there has been increased focus on the importance of non-pharmacological interventions, such as cognitive stimulation, which has been shown to improve cognitive function and psychological wellbeing, as well as the overall quality of life of families (Pickett et al., 2018; Atay and Bahadır Yılmaz, 2024; Paggetti et al., 2024; Bertrand et al., 2023).

Many interventions have been implemented in recent decades, but cognitive stimulation (CS) has shown the strongest evidence for improving cognitive functions among various psychosocial approaches (McDermott et al., 2019; Desai et al., 2024). CS is a widely used non-pharmacological intervention aimed at improving or maintaining cognitive functions, such as memory, attention, language skills, and executive functions. It consists of thematic activities designed to exercise and train various cognitive areas through exercises, including more playful tasks like categorization, word association, discussion of current events, problem-solving, selective attention, and discussions (Woods et al., 2012; Spector et al., 2003). Sessions are led by an experienced facilitator who can adapt activities flexibly based on the patient’s interests, needs, cultural contexts, and cognitive/sensory abilities. The main goal of CS is to slow cognitive decline and improve the psychological wellbeing of participants. The therapeutic benefits of CS are well-documented, with studies highlighting its ability to improve cognitive skills such as memory and attention, as well as reduce the psychological symptoms of dementia (Capotosto et al., 2017; Gibbor et al., 2021a; Gibbor et al., 2021b; Saragih et al., 2022), in addition to having potential effects on brain physiology (Liu T. et al., 2021). Furthermore, CS is considered beneficial for caregivers as it reduces their stress and improves their satisfaction in providing care (Spector et al., 2003; Orrell et al., 2017; Lauritzen et al., 2023). Additionally, CS is the only non-pharmacological intervention recommended by the National Institute for Health and Care Excellence (NICE) to improve cognition, independence, and wellbeing in people with dementia (NIHCE, 2018; Cartabellotta et al., 2018).

Despite the evidence supporting its effectiveness (Lobbia et al., 2019; Pike et al., 2024), the World Alzheimer’s Report 2022 has recommended further research and the global implementation of CS, particularly in terms of user satisfaction and long-term effectiveness (Gauthier et al., 2022).

CS was initially developed as an in-person intervention. However, the COVID-19 pandemic has underscored the need for remote therapeutic solutions, both to limit in-person healthcare access and to ensure continuity of care for patients with dementia (Liu K. Y. et al., 2021; Bethell et al., 2021; O’Connell et al., 2021; Santini et al., 2022). Consequently, the pandemic led to an increased use of technology for the delivery of remote healthcare services via videoconferencing platforms. In this context, the use of technology for delivering psychosocial interventions for people with dementia has become a rapidly expanding field (Cuffaro et al., 2020; Fisher et al., 2023).

Reviews and meta-analyses examining the delivery of psychosocial interventions for people with dementia and their caregivers have demonstrated that virtual delivery was feasible and resulted in comparable outcomes to in-person delivery (Burton and O’Connell, 2018; Perkins et al., 2022; Fisher et al., 2024; Lai et al., 2020). Specifically, recent studies have explored the effectiveness of online interventions, with findings suggesting that video-conferenced CS can yield benefits similar to face-to-face delivery (Spector et al., 2024).

However, the inherent limitations of the “internet-based” modality must be acknowledged, including access to digital technology, the need for specific skills, ethical and security issues, as well as skepticism toward digital environments (Pywell et al., 2020; Yi et al., 2021). Despite these barriers, the benefits for individuals with reduced mobility, transportation difficulties, or those living in areas far from care centers are undeniable (Cuffaro et al., 2020; Adams et al., 2020; Angelopoulou et al., 2022).

In Italy, the development of telemedicine is relatively recent, with disparities among regions in the provision of related services (Maresca et al., 2020). In this context, considering its potential within the National Health Service, it remains crucial to assess the effectiveness and applicability of telemedicine interventions for patients with dementia, taking into account their specific needs and potential technological barriers. This pilot study aims to evaluate the feasibility and effectiveness of a CS intervention for dementia patients, contributing to the development of more accessible and personalized therapeutic strategies.

## 2 Materials and methods

### 2.1 Participants

Subjects were recruited at the Center for Cognitive Disorders and Dementia in Pisa. The inclusion criteria were as follows: they had to (1) meet the DSM-5 criteria for dementia, classified as Major Neurocognitive Disorder, as assessed by a trained clinician, following a comprehensive neurological and neuropsychological evaluation for the diagnosis (American Psychiatric Association, 2013), (2) be classified as having mild or moderate dementia based on the Clinical Dementia Rating Scale (Morris, 1993), (3) do not have auditory or visual impairments, as assessed through clinical anamnesis, in order to engage in conversations and interpret visual materials and (4) able to communicate verbally in Italian. For those engaging in the videoconferencing mode, participants were required to have a desktop computer and be capable of conducting a video call using the selected platform for the sessions, if necessary, with the assistance of a caregiver only in case of technical difficulties or to verify a good connection, without interfering with the activities.

The study involved 19 dementia patients (Clinical Dementia Rating = 1 or 2), with 12 participating in in-person treatment and seven engaged remotely. Over the course of eight weekly 1 h sessions, patients participated in various cognitive activities, including memory, attention, and problem-solving exercises, guided by a clinical psychologist.

The “Internet-based” sessions utilized the televisita.sanita.toscana.it, a platform provided by the national healthcare system, while the “On-site condition” meetings were held at the Neurology Unit, Cognitive Disorders and Dementia Centre, Pisa University Hospital. All participants attended at least one introductory meeting; for those engaging in videoconference sessions, an additional “computer literacy” meeting was conducted to ensure proper use of the online platform. During this introductory meeting, it was verified that participants had the digital skills necessary for carrying out the activities (connection to the platform, turning on the microphone and camera, interaction, etc.). The sessions are synchronous and not recorded. During the videoconference sessions, in case of any difficulties with platform usage or connectivity, instant chat support, telephone contact, and an email address were provided as points of reference.

All participants provided written informed consent for participation, which included information on privacy and the handling of sensitive data. The study protocol was approved by the Regional Ethical Committee for Clinical Experimentation (Comitato Etico di Area Vasta Nord Ovest - CEAVNO) for the publication of aggregated, anonymized data collected from medical records without experimental procedures. The study adhered to the ethical guidelines outlined in the 1975 Declaration of Helsinki.

### 2.2 Cognitive stimulation protocol

This protocol consists of eight weekly, 1 h individual sessions of CS for persons with dementia. Every session is led by a clinical psychologist, who was not involved in the assessment, with professional qualifications and expertise in neurodegenerative diseases. The meeting focuses on engaging the participant in cognitive activities designed to stimulate memory, attention, and problem-solving skills, as well as teaching compensatory strategies useful in daily life. The exercises are carefully calibrated to match the participant’s cognitive abilities and residual capacities, ensuring that they are appropriately challenging yet achievable. Each session begins with a brief and friendly greeting, designed to establish rapport and create a comfortable environment for the participant. Following the greeting, a short orientation exercise is conducted, where the participant is prompted to engage with details about the current day, date, time, and place. This helps stimulate attention and reinforces their temporal and spatial awareness. After the orientation, the session moves into focused exercises aimed at sharpening attention. These tasks may include simple visual or auditory attention tasks tailored to the participant’s cognitive level.

The main part of the session introduces the main theme of the meeting; the eight topics are summarized in Table 1. The exercises are adjusted in difficulty depending on the individual’s capabilities, with the goal of stimulating cognitive processes without causing frustration. Activities may involve recalling past events, solving simple problems, or engaging in discussions that require reasoning and attention. At the end of the main activity, the psychologist helps the participant generalize the cognitive skills practiced during the session, linking them to everyday activities or situations. This reflective step encourages the participant to recognize how these cognitive exercises can relate to their daily life and routines.

**Table 1:** Themes covered eight sessions of cognitive stimulation program every week.

Meeting	Topic
1	*Short-term memory* Immediate recall of verbal and visuospatial material.
2	*Working memory* Immediate recall of verbal and visuospatial material with active manipulation (e.g., “Put in order”)
3	*Long-term memory: encoding strategies* Delayed recall of previously analyzed verbal and visuospatial material using memorization strategies.
4	*Categorization* Use of verbal and visuospatial material for categorization exercises.
5	*Semantic memory* Delayed recall of semantically structured verbal and visual material.
6	*Language: morphosyntactic structure* Activities using verbal and visual material for naming, repetition, and sentence construction.
7	*Language: lexical access* Activities using verbal and visual material for verbal fluency tasks.
8	*Generalization of learned strategies in daily life* Application of memorization strategies useful for remembering appointments, shopping lists, phone numbers, etc.

Each session concludes with a brief discussion of how the participant felt about the activities and any observations about their progress. The psychologist provides positive reinforcement and ends the session with a warm farewell, leaving the participant with a sense of accomplishment.

The protocol has been adapted to an online format, maintaining the characteristics of in-person execution. The sessions were conducted via videoconferencing platforms of the public health system. In this mode, both verbal and visual materials are used. Verbal instructions and discussions remain central to the session, while visual aids (e.g., images, documents, or interactive exercises) are shared through the screen-sharing function, ensuring that the participant can fully engage with the content.

### 2.3 Assessment

Assessments were conducted using the Mini-Mental State Examination (MMSE; Folstein et al., 1975), Activities of Daily Living (ADL; Katz et al., 1963), and Instrumental Activities of Daily Living (IADL; Lawton and Brody, 1969) at the start (T0) and end (T1) of the intervention.

Visual-Analog Scale (VAS) scale is widely employed in subjectively assessing various variables (Voutilainen et al., 2016): specifically, a vertically oriented VAS ranging from 0 to 100 was utilized. Participants were asked to rate aspects proposed on this continuum, for example: “How useful do you feel participating in these sessions was for you from 0 (none) to 100 (very extensive)?” (Table 2).

**Table 2:** Crucial facets of the patients and caregiver experience linked to the cognitive stimulation sessions assessed using a visual-analog scale (VAS) at the end (T1) of the program.

Questions in visual-analog scale (VAS) for patients
How useful do you feel participating in these sessions was for you?
How enjoyable do you feel participating in these sessions was for you?
Questions in visual-analog scale (VAS) for caregivers
How useful do you feel your family member’s participation in these sessions was?
How enjoyable do you feel your family member’s participation in these sessions was?

At the end of the program, both patients and caregivers were invited to indicate their level of agreement or disagreement with specific statements by completing a 4-point or 3-point Likert scale (Tables 3, 4). In addition, the overall participation rate, dropout rate, and completion of outcome measures were taken into consideration.

**Table 3:** Research questions on the acceptability and effectiveness posed to patients with Likert scale at the end of the cognitive stimulation program.

Acceptability and effectiveness questions for patients
Did you feel comfortable with your group facilitator? *(Very - Quite - A little - Not at all)*
Was it useful to complete “homework” assignments? *(Very - Quite - A little - Not at all)*

**Table 4:** Research questions on the acceptability, feasibility, and effectiveness posed to caregivers with Likert scale at the end of the cognitive stimulation program.

Acceptability, feasibility, and effectiveness questions for caregivers
Before the session or while waiting in the waiting room, what was your family member’s reaction? *(positive - neutral - negative)*
After the session, what was your family member’s reaction? *(positive - neutral - negative)*
How did your family member react to having to complete “homework” assignments? *(positive - neutral - negative)*
Do you think the frequency of the sessions was appropriate? *(positive - neutral - negative)*
Were the sessions in line with your expectations? *(positive - neutral - negative)*
Did you feel supported by the service (for example, did you feel less alone)? *(positive - neutral - negative)*
Did you perceive any benefits or improvements? *(positive - neutral - negative)*
Would you like another cycle of sessions to be offered? *(positive - neutral - negative)*
Did you feel welcomed and given enough space for your questions? *(positive - neutral - negative)*
Was it challenging for you to help your family member with the exercises at home? *(positive - neutral - negative)*

### 2.4 Statistical analysis

To describe the demographic and clinical characteristics of the study population and aspects of experience related to the CS program, investigated using a Visual-Analog Scale, descriptive parameters were calculated, including the mean and standard deviation (mean ± SD).

Differences between internet-based and on-site conditions groups for age, education, gender and type of dementia have been evaluated using for quantitative variables, unpaired *t-*tests or the nonparametric Mann–Whitney Rank Sum Test; for categorical variables, Chi-squared tests or Fisher’s exact method was applied. Shapiro–Wilk test was considered to test the normal distribution of quantitative variables.

For cognitive function (MMSE), daily living activities (ADL and IADL), and dementia severity (CDR) scores, a two-way analysis of variance (ANOVA) for repeated measures (RM) was performed, considering both factor “group” (on-site condition or internet-based) and factor “time” (at beginning, T0, or at the end, T1), with *post-hoc* analysis Holm-Sidak method. Effect sizes (ES) were calculated using Cohen’s d statistic.

Statistical analyses were performed using statistical software package Sigma Stat 4.0; statistical significance was assumed for a *p* < 0.05.

## 3 Results

Of the 19 subjects, *n* = 12 followed the program in “on-site conditions,” while *n* = 7 were in “internet-based” group. The two groups exhibited similarity in age, education, gender and type of dementia (*p* > 0.05 for all *p*-value; Table 5); specifically, the mean age was around 65 years, with 12 years of education.

**Table 5:** Demographic characteristics of participants in on-site condition and internet-based [data are expressed as n, *n* (%), mean ± SD].

	Total (*n* = 19)	On-site condition (*n* = 12)	Internet-based (*n* = 7)	*p*-value
Age, years (means ± SD)	64.7 ± 10.2	65.2 ± 10.6	64 ± 10.5	0.82
Education (means ± SD)	11.9 ± 4.3	11.7 ± 4.5	12.1 ± 4.3	0.85
Sex, women (*n*, %)	51 (69.9)	6 (50)	4 (57.1)	0.86
Type of dementia, *n* (%)				
Alzheimer disease	13 (68.4)	7 (58.3)	6 (85.7)	0.22
Parkinsonism-related dementia	6 (31.6)	5 (41.7)	1 (14.3)	

No differences were found at baseline in either global cognitive functioning measured by the MMSE, or in the ability to perform activities of daily living (ADL) and instrumental activities of daily living (IADL). These parameters remained stable throughout the duration of the intervention for both groups (Table 6). The severity of dementia and the degree of cognitive impairment in individuals also remained unchanged at the end of the CS program (Table 6), with a small effect size on dementia status (ES = 0.15 for MMSE; ES = 0.14 for CDR).

**Table 6:** Descriptive statistics (mean ± standard deviation) and statistical analysis for MMSE, ADL, IADL, and CDR scores for two groups, on-site condition and internet-based, at beginning (T0) and at the end (T1) of cognitive stimulation program.

	On-site condition (T0)	On-site condition (T1)	Internet-based (T0)	Internet-based (T0)	*p*-value time	*p*-value group	*p*-value time*group
MMSE (means ± SD)	22.9 ± 4.0	23.9 ± 4.1	22.6 ± 4.1	22.1 ± 4.0	0.935	0.545	0.218
ADL (means ± SD)	5.6 ± 0.9	5.5 ± 1.0	5.8 ± 0.4	5.8 ± 0.4	0.461	0.412	0.461
IADL (means ± SD)	5.9 ± 2.4	5.6 ± 2.5	6.7 ± 1.4	6.6 ± 1.4	0.070	0.411	0.605
CDR (means ± SD)	0.9 ± 0.5	0.9 ± 0.5	1.1 ± 0.6	1.1 ± 0.6	1.000	0.508	1.000

Results provide an overview of cognitive function (MMSE), daily living activities (ADL and IADL), and dementia severity (CDR) across the study population toward two-way ANOVA RM. No significant differences were observed in the analyzed variables for the time factor (T0 vs. T1), the group factor (on-site condition vs. internet-based), or the interaction between the two factors (time*group).

**Figure 1:**
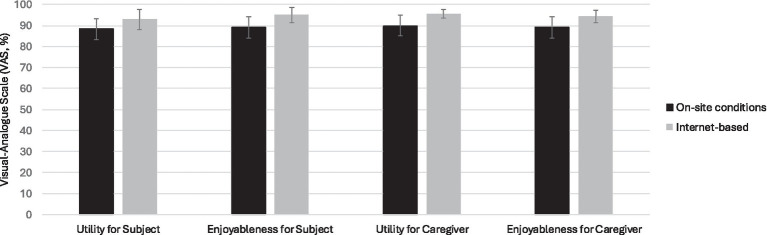
Aspects of subjects and caregiver experience related to investigated using a visual-analog scale in internet based and on-site conditions at the and end (T1) of the program. Mann–Whitney rank sum test on-site condition and internet-based for “Utility for Subject and Caregiver,” “Enjoyableness for Subject and Caregiver” was performed with *p* > 0.05 for all *p*-values. Error bars = s.e.m.

Both patients and caregivers reported high levels of satisfaction with the intervention, providing positive feedback regarding the utility, enjoyment, and engagement in the sessions. At the end of the CS program, the subjects reported an overall utility of 90% and an enjoyment of 91.3%; caregivers’ assessments aligned with a mean score on the VAS scale of 92.1 and 91%, respectively. No statistically significant differences were observed between the two modalities (*p* > 0.05 for all *p*-values; Figure 1). Specifically, subjects found the relationship with the psychologist to be comfortable (94.7% of subjects responded “very” on the Likert scale), and they considered the assigned homework useful (52.6% responded “very” and 47.4% responded “quite” on the Likert scale) (Table 3). The average adherence rate was 92.86% for the “internet-based” group and 97.92% for the “on-site conditions” group. Specifically, the absences were due to personal and health-related reasons; only two patients were unable to attend a session due to connection issues. No dropouts occurred, and all outcome measures were completed by every participant.

Caregivers reported positive feelings regarding the family member’s reactions before (89.5% positive responses), after the sessions (100% positive responses), and in relation to the homework assignments (78.9% positive responses). Caregivers highlighted a high degree of appropriateness for the intervention regarding session frequency (89.5%) and alignment with their expectations (100%). The program was also found to be effective in providing support and welcoming the caregivers (100%), as well as fostering perceived benefits and improvements (89.5%). For half of the caregivers, it was challenging to assist their family member with the exercises at home (50%). Overall, all caregivers interviewed expressed great enthusiasm for the series of sessions and requested the implementation of a second cycle of meetings. Such feedback, recorded at the end of the CS program using the Likert scale (Table 4), was consistent in both the “on-site condition” and “internet-based” modalities.

## 4 Discussion

The global prevalence of dementia, combined with its profound impact on patients and caregivers, has stimulated the search for interventions aimed at counteracting cognitive decline and improving quality of life. This study fits within this framework, documenting the effectiveness of CS in maintaining cognitive functions. Specifically, by incorporating both in-person and online modalities, this research evaluates the adaptability and feasibility of CS in different settings, assessing technological barriers in addition to the perceived benefit for caregivers.

Before the pandemic, few studies on the use of digital technologies in dementia care had already highlighted that, despite difficulties, offering online interventions to people with dementia was both possible and beneficial (Burton and O’Connell, 2018; World Health Organization, 2021; Dal Bello-Haas et al., 2014). Subsequently, with the onset of the pandemic, an increasing number of studies have corroborated these findings (Fisher et al., 2024; Gonzalez-Moreno et al., 2022; Spector et al., 2024; Santini et al., 2022).

The results indicate that CS, delivered both in-person and via videoconferencing, is feasible and well-received by both patients and caregivers. The absence of statistically significant differences between the two groups in terms of cognitive functionality (MMSE scores) and daily activities (ADL and IADL scale scores) suggests that remote delivery is as effective as traditional in-person sessions. Although the MMSE may have limited sensitivity to subtle changes over time, particularly in critical age groups from 70 to 94 years (Aiello et al., 2022), its widespread use and reliability as a cognitive screening test make it a valuable tool. In our sample, with a mean age of approximately 65 years, this effect should be reduced, making the MMSE a valid tool in the context of the present pilot study. Both modalities achieved high participation and completion rates, reinforcing the adaptability of CS in different delivery formats.

Participants and caregivers reported high levels of satisfaction, with average scores above 90% in various domains, including perceived utility, enjoyment, and engagement. The therapeutic relationship with the psychologist was particularly appreciated, with over 94% of participants expressing comfort in this interaction. Caregivers also provided extremely positive feedback regarding the frequency of the program, alignment with expectations, and the support received. Despite some minor difficulties, such as assistance with homework tasks (reported by 50% of caregivers), the overall enthusiasm for the intervention highlights its potential acceptability, feasibility, and effectiveness for wider implementation.

The proposed cycle of sessions, in its brevity, includes activities designed to train various cognitive functions, such as memory, attention, and language, also within the context of daily use, promoting greater practical applicability. Additionally, it offers considerable flexibility, as it can easily be adapted to the specific needs of participants. The cycle can be repeated to consolidate the skills learned or to adjust the difficulty level. The exercises can be varied in their mode of delivery based on the participants’ remaining abilities. For example, for those with higher language skills, activities can emphasize verbal components such as storytelling or word games. Conversely, for individuals who prefer visuospatial tasks, exercises like image recognition or spatial orientation activities can be proposed. This personalization helps optimize engagement and the effectiveness of the intervention, making it accessible and meaningful for a wide range of participants.

The study results align with previous research highlighting the psychological and social benefits of CS (Cicerone et al., 2000; Clare et al., 2019), also in comparison to control groups (Gonzalez-Moreno et al., 2025). Moreover, the consistency of results across delivery modalities supports the idea that technological adaptation does not compromise the protocol’s effectiveness. This makes internet-based CS a service that complements face-to-face delivery, available at clinics, with the added advantage of increasing accessibility for those unable to attend in-person sessions due to various clinical, mobility, personal, transportation, or family management restrictions.

Low levels of digital literacy may represent a barrier for older adults (Fisher et al., 2023; Tan et al., 2020), as videoconference calls can be complex to set up and require high digital skills. As with previous studies, this one addressed this issue by providing preliminary support sessions for participants and families to resolve potential difficulties.

Caregivers spontaneously reported mood improvements following participation in CS sessions. Additionally, participants in the internet-based modality reported greater confidence and comfort with using digital technologies, such as videoconferencing calls.

Overall, this pilot study highlights the feasibility, acceptability, and effectiveness of CS interventions delivered both in-person and online. The primary advantage lies in its adaptability, offering an inclusive approach to dementia care that addresses logistical challenges such as mobility and geographic access. The positive reception of the intervention by patients and caregivers, along with comparable results across modalities, underscores its potential for broader application in clinical practice.

Feedback indicated that remote participation was well-received, with no significant technological issues or barriers to interaction reported. Furthermore, participants from both groups demonstrated similar levels of participation and interaction, supporting the feasibility of remote treatment as a valid alternative to in-person sessions. These results suggest that the intervention can be successfully delivered remotely without compromising patient engagement or care outcomes.

However, the study has some limitations. The small sample size limits the generalizability of the results, and the short duration of the intervention does not allow for conclusions on its long-term effectiveness. Additionally, although caregivers provided positive feedback, their role in supporting home exercises requires further exploration to address potential difficulties and improve their involvement in relation to caregiver burden outcomes. Additional objective measurements to increase sensitivity in detecting changes over time and to assess further outcomes related to the neuropsychiatric symptoms of patients would be useful to confirm the positive perception reported by their family members. While acknowledging that the lack of a control group and the absence of randomization represent methodological limitations, it is important to emphasize the focus on exploring feasibility and gathering preliminary data for future, broader, and more rigorous research. Moreover, in a “real world” context, the uncontrolled nature of the intervention allows for the observation of individual dynamics in situations that are closer to everyday clinical practice.

Future research should focus on expanding the intervention to include a larger and more diverse sample and extending the program duration to assess sustained benefits over time. Investigating digital literacy and accessibility could inform strategies to reduce barriers to remote delivery. Additionally, exploring the integration of personalized caregiver support within the CS framework could enhance the overall impact of the intervention.

## 5 Conclusion

In conclusion, this study provides valuable evidence supporting the usefulness of CS in dementia care and highlights the potential of remote modalities to expand access to effective, person-centered interventions. Further research and refinements are essential to optimize delivery and maximize benefits for patients and caregivers.

## Data Availability

The raw data supporting the conclusions of this article will be made available by the authors, without undue reservation.
